# Medical Literature in the South

**Published:** 1884-09-20

**Authors:** 


### MEDICAL LITERATURE IN THE SOUTH.

Since the late war there has been a perceptible increase of interest in the Profession and its advancement in the Southern States. We are gratified to note this, and to be able to state that, while there is yet too much indifference in the matter of communicating facts and cases for the journals, there is an improvement. There are those who think, and who have the ambition and energy to write their thoughts; and we are proud to say that the physicians of our section are, in respect to their ability and attainments, fully up to the standard that exists elsewhere. In fact, we think we can truthfully say that they are more practical on many points; particularly may this be said in regard to the treatment of fevers, and in the discovery and use of new and indigenous remedies,

Among the many agents which, in a comparatively recent period, have thus been brought to the notice of the Profession by Southern physicians may be mentioned, as important, the following: *veratrum viridi*, *gossipii radices cortex*, viburnum prunifolium, gelsemium, etc. Some of these are very popular, and in general use throughout the country.

We are thankful to those of our readers who have contributed to our pages in the past, and take this opportunity to say that we hope that more of our subscribers will take upon themselves this task and assist us in developing and bringing to light the medical knowledge of our section. We prefer brief and practical articles Papers designed for any particular month should reach us by the first of the month, about which time the original department of our journal goes to press. We are pleased to receive useful formulæ, such as have been tested in practice and found to be reliable and safe.

